# Intravenous lipid emulsion for the treatment of poisonings in 313 dogs and 100 cats (2016–2020)

**DOI:** 10.3389/fvets.2023.1272705

**Published:** 2023-09-28

**Authors:** Carina Markert, Romy Monika Heilmann, Dschaniena Kiwitz, René Doerfelt

**Affiliations:** ^1^Veterinary Clinic for Small Animals, Hofheim, Germany; ^2^Department for Small Animals, College of Veterinary Medicine, University of Leipzig, Leipzig, Germany; ^3^LMU Small Animal Clinic, Ludwig-Maximilians-Universität München, Munich, Germany

**Keywords:** intralipid, log P, intoxication, dogs, cats

## Abstract

**Introduction:**

The aim of this retrospective study was to analyze the effect and potential adverse effects of intravenous lipid emulsion (ILE) in poisoned dogs and cats over a 5 years period.

**Methods:**

Medical records of 313 dogs and 100 cats receiving ILE between 2016–2020 were analyzed for suspected toxicant, clinical signs, ILE dosages and frequency, the effect and adverse effects of ILE, and patient outcome.

**Results:**

Dogs and cats were poisoned with mostly unidentified toxicants (48%), rodenticides (8%), recreational drugs and nuts (7% each) and other toxicants. Clinical signs included neurologic deficits (63%), cardiovascular signs (29%), thermoregulation (21%) or gastrointestinal abnormalities (17%). Treatment with ILE was initiated within a median of 6.0 h (1.0–91.0 h) after poisoning. Dogs and cats received a total amount of median 8.0 mL/kg (1.5–66.6 mL/kg) and 15.8 mL/kg (1.8–69.4 mL/kg) ILE, respectively. A positive effect was observed in 74% of the patients, whereas clinical signs worsened in 4% of the patients after ILE administration. No subjective effect was detected in 22% of the patients. Suspected or possible adverse effects of ILE occurred in 6% of the patients, including neurological signs (temporarily reduced consciousness and ataxia), bradycardia, hyperthermia, vomiting, diarrhea, respiratory distress, worsening of the general behavior, facial swelling, and thrombophlebitis. The overall survival rate was 96%. One dog who potentially experienced adverse events was euthanized.

**Conclusion:**

ILE treatment was successful in most patients but can be associated with adverse effects. Administration of ILE should be carefully selected on an individual basis after weighing the possible benefits against potential adverse effects.

## 1. Introduction

Poisoning is a common cause for dogs and cats to be presented to the veterinary emergency service. In addition to symptomatic treatment and decontamination attempts, intravenous lipid emulsions (ILE) are commonly administered to eliminate lipophilic toxicants from their sites of action. Several case reports have been published, but only one prospective clinical study (1) apart from an investigation in laboratory animals (2) has been performed to evaluate the effect of ILE in dogs and cats.

The first studies in the 1970s and 1980s described the effects of ILE on the pharmacokinetics of chlorpromazine (3) and ciclosporin in rabbits (4) and phenytoin in rats (5). Connecting to this experimental success with laboratory rats (6), protective effects of lipid emulsions were also observed in dogs (7). The reversibility of bupivacaine-induced cardiotoxicity and improvement of myocardial function was demonstrated in 6 dogs (7). Another case report (8) described the successful use of ILE in a Jack Russell terrier puppy with confirmed moxidectin poisoning. In a case series of 20 cats that had inadvertently received a parenteral 20-fold overdose of ivermectin for the treatment of ear mites, it was hypothesized that ILE reduces the plasma concentration of the toxin, especially in the absence of fat reserves and a low body condition score, provided that ILE therapy is initiated early in asymptomatic patients and is administered continuously (9). Clarke et al. also concluded that the plasma toxin concentration of ivermectin increases after ILE as well (10). It has been proposed that, according to the lipid shuttle theory, the toxic substance is redistributed by the lipid contained in the plasma and metabolized or excreted by the liver or kidneys and thus prevented from reaching the target tissues. Further case reports support this hypothesis or describe the beneficial effects of ILE on mitochondrial recovery and direct inotropy by increasing the calcium concentration within cardiac myocytes (11–14). In a prospective clinical study, faster positive outcomes were achieved with ILE treatment (0.25 mL/kg/min over 60 min) compared to the isotonic saline administration in the control group based on a clinical classification system for cats with permethrin intoxication (1). This led numerous authors to conclude that ILE is a useful adjunctive therapy in the treatment of various poisonings (15–17). However, multiple adverse effects have been reported in human (HM) and veterinary medicine (VM). These include cardiovascular (18, 19) and pulmonary complications (20, 21), including ventilatory/perfusion disturbances (22, 23), hematologic abnormalities (24, 25), neurologic signs (26–28), including a comatose state (29), and hypertriglyceridemia (30, 31) with lipid overload syndrome after a too rapid infusion (32, 33). Less frequently, corneal lipidosis (34), acute anaphylactoid reactions (35, 36), and in HM, acute kidney injury (37), metabolic acidosis (38), or hypercoagulability associated with total parenteral nutrition (39) have been described as adverse effects of ILE.

Given the current knowledge gap on ILE therapy in VM, the objective of this retrospective study was to analyze the use of ILE in a large cohort of dogs and cats with confirmed or suspected poisonings, including the effects, adverse effects, applied dosages and outcomes of ILE. These cases were presented at the emergency service of a transregional veterinary referral center over a 5 years period. We hypothesized that the administration of ILE presents a safe treatment option and leads to a reduction in clinical signs of poisonings with lipophilic toxicants.

## 2. Materials and methods

Electronic medical records (MR) of the emergency service of the transregional veterinary referral center in Germany were searched for “poisoning”/“intoxication,” “intravenous lipid infusion,” or the brand of the available intravenous lipid infusion “Lipofundin” between 2016–2020.

Meeting these inclusion criteria, 416 patients were identified. Three patients with underlying primary neurologic diseases diagnosed in the course of the disease or with presumed poisoning but without evidence of poisoning based on the patient history or physical examination were excluded from the analysis. Thus, a total of 413 animals (313 dogs and 100 cats) with oral (*n* = 405), transdermal (*n* = 6), or oral and transdermal (*n* = 2) poison exposure that received ILE (Lipofundin MCT/LCT 20%, B. Braun, Melsungen, Germany) were included in the retrospective evaluation.

Information retrieved and analyzed from the electronic MR were: (1) demographic data; (2) clinical signs at admission and during hospitalization; (3) the toxicant (confirmed via toxicological analysis of urine, feed reserve/leftovers, or stomach contents or clinicopathologic evidence; or suspected); (4) times from poisoning and presentation at the clinic to ILE application; (5) dosages of ILE bolus and continuous rate infusion (CRI); (6) duration of application, frequency, and the total amount of ILE administered to the patient (ILE I, first application; ILE II, second application; ILE III, third application; ILE IV, fourth application; ILE V, fifth application; ILE VI, sixth application); (7) clinicopathologic findings, including hematology (ProCyte Dx, Idexx), biochemistry profile (Integra 400 plus, Roche Cobas), and plasma ionized calcium concentration (I-Stat, Scil); (8) the effect and adverse effects of ILE administration; (9) outcome, (10) disease course with any adverse effect, and (10) duration of hospitalization.

Patient data were recorded using a commercial software program (Excel, Microsoft Office 2016, Microsoft Corporation, Redmond, WA, United States) and were statistically analyzed (Prism 5, GraphPad, San Diego, CA, United States). Continuous data were evaluated for normal distribution using the D’Agostino & Pearson normality test. Summary statistics were reported as medians and ranges (non-parametric data). The proportion of male and female animals as well as that of decontaminated and not decontaminated animals with or without adverse effects was analyzed using the Fisher’s exact test. ILE doses between dogs and cats with or without adverse effects and the times to improvement between dogs and cats were compared using the Mann–Whitney U test. The correlation between time to improvement and total ILE dose was analyzed using the Speaman correlation. Statistical significance was set at *p* < 0.05.

## 3. Results

### 3.1. Demographic data

Over the 5 years period, 313 dogs and 100 cats that presented with confirmed or suspected poisoning were treated with intravenous lipid emulsion (ILE). Predominant breeds in dogs were crossbreed (86/313, 27.5%) and Labrador Retriever (47/313, 15.0%), followed by Golden Retriever (12/313, 3.8%), Australian Shepherd (11/313, 3.5%), Jack Russell terrier (11/313, 3.5%), and Chihuahua (10/313, 3.2%). In cats, the Domestic Shorthair (76/100, 76.0%) was the predominant breed, followed by the British Shorthair (8/100, 8.0%) and crossbreed (5/100, 5.0%). Other breeds presenting with less than 3.0% of the species were not listed.

Male dogs (95/313, 30.4% intact and 63/313, 20.1% neutered) and cats (41/100, 41.0% neutered and 8/100, 8.0% intact) were represented with similar frequency as female dogs (80/313, 25.6% intact and 74/313, 23.6% spayed) and cats (40/100, 40.0% spayed and 10/100, 10.0% intact; *p* = 0.908). Sex was not documented in the electronic MR for one dog and one cat. The median age of all dogs included in the study was 3.6 years (0.2–14.1 years) and of all cats was 3.5 years (0.2–16.0 years). The median weight was 21.9 kg (1.3–64.0 kg) in dogs and 4.2 kg (0.8–8.5 kg) in cats.

### 3.2. Patient history and clinical presentation

Clinical signs became apparent within a median of 2.0 h (0.1–48.0 h) in dogs (*n* = 238) and 2.0 h (0.1–72.0 h) in cats (*n* = 40) after suspected or witnessed toxicant ingestion or contact. Presentation at the emergence service of the clinic was within a median of 5.0 h (0.3–50.0 h) in dogs (*n* = 244) and 5 h (1.0–90.0 h) in cats (*n* = 43).

Clinical signs at the time of hospital admission were predominantly neurologic signs (83.3%), followed by an altered general behavior (35.4%; dogs: *n* = 109, 34.8%; cats: *n* = 37, 37.0%), cardiovascular signs or dehydration (28.8%; dogs: *n* = 89, 28.4%; cats: *n* = 30, 30.0%), altered thermoregulation (21.5%), and gastrointestinal signs (16.5%; dogs: *n* = 53, 16.9%; cats: *n* = 15, 15.0%). Respiratory signs (4.6%; dogs: *n* = 14, 4.5%; cats: *n* = 5, 5.0%), evidence of hemorrhage (0.5%; dogs: *n* = 1, 0.3%; cats: *n* = 2, 1.0%), and allergic reactions (0.2%; dogs: *n* = 1, 0.3%) were less common. In a few patients (dogs: *n* = 12, 3.8%; cats: *n* = 1, 1.0%), no clinical signs were observed (Table 1).

**Table 1:** Clinical signs at the time of presentation at the emergency service of the clinic in 313 dogs and 100 cats with confirmed and suspected poisoning treated with intravenous lipid emulsion (ILE).

Clinical signs	*n* (dogs)	% (dogs)	*n* (cats)	% (cats)	*n* (total)	% (total)
Neurologic signs	255	81.5	89	89.0	344	83.3
Altered general behavior	109	34.8	37	37.0	146	35.4
Cardiovascular/hydration changes	89	28.4	30	30.0	119	28.8
Altered thermoregulation	46	14.7	41	41.0	87	21.1
Gastrointestinal signs	53	16.9	15	15.0	68	16.5
Respiratory signs	14	4.5	5	5.0	19	4.6
Evidence of hemorrhage	1	0.3	1	1.0	2	0.5
Allergic reactions	1	0.3	–	–	1	0.2
No clinical signs	12	3.8	1	1.0	13	3.1

*n*, number of dogs or cats showing the respective clinical signs.

### 3.3. Toxicants

Strong indication or confirmation of poisoning was based on the patient history, clinical signs, observation of the exposure or ingestion of the toxicant, results of toxicological analysis, and/or detection of the toxicant in gastric contents. Toxicologically confirmed agents were theobromine, clozapine, caffeine, parathion/sulfotep, and alpha-chloralose, for which qualitative analysis (no quantification) was performed. However, the toxicant remained unidentified in almost half of the patients (200/413, 48.4%; dogs: *n* = 142, 45.4%; cats: *n* = 58, 58.0%). Poisonings with one or more than one toxicant were strongly suspected or observed with similar frequency (213/413; 51.6%). Poisonings with tremorgenic mycotoxins (43/413; 10.4%; only dogs: *n* = 43, 13.7%) and rodenticides (33/413, 8.0%; dogs: *n* = 12, 3.8%; cats: *n* = 21, 21.0%) were more frequently reported than those with recreational drugs (30/413, 7.3%). These were followed in the frequency of occurrence by ingested toxic plants (23/413, 5.5%; dogs: *n* = 22, 7.0%; cats: *n* = 1, 1.0%), food leftovers/kitchen waste (23/413, 5.5%; only dogs: *n* = 23, 7.3%), potentially tremorgenic mycotoxins (22/413, 5.3%; only dogs: *n* = 22; 27.0%), chemicals (21/413, 5.1%; dogs: *n* = 13, 4.2%; cats: *n* = 8, 8.0%), antiparasitics (16/413, 3.9%; dogs: *n* = 10, 3.2%; cats: *n* = 6, 6.0%), medication (12/413, 2.9%; dogs: *n* = 8, 2.6%; cats: *n* = 4, 4.0%), feces- or manure-associated poisonings (10/413, 2.4%; only dogs: *n* = 10, 3.2%), nuts (6/413, 1.5%; only dogs: *n* = 6, 1.9%), and molluscicides (5/413, 1.2%; only dogs: *n* = 5, 1.6%). Some dogs (*n* = 9, 2.9%) were poisoned with more than one toxicant in the same or even different toxicant group (Table 2).

**Table 2:** Summary of the toxicants confirmed or suspected in 313 dogs and 100 cats treated with intravenous lipid emulsion (ILE).

Toxicant	*n* (dogs)	% (dogs)	*n* (cats)	% (cats)	*n* (total)	% (total)
**Total**	**313**	**100**	**100**	**100**	**413**	**100**
**Unknown**	**142**	**45.4**	**58**	**58.0**	**200**	**48.4**
**Tremorgenic mycotoxins**	**43**	**13.7**	**–**	**–**	**43**	**10.4**
Walnut* (penitrem A, roquefortine)	21	6.7	–	–	21	5.1
Garbage	11	3.5	–	–	11	2.7
Tainted meat/feed	5	1.6	–	–	5	1.2
Compost	5	1.6	–	–	5	1.2
Other mycotoxin	1	0.3	–	–	1	0.2
**Rodenticides**	**12**	**3.8**	**21**	**21.0**	**33**	**8.0**
Alpha-chloralose	7	2.2	16	16	23	5.6
Coumatetralyl or unknown	4	1.3	4	4.0	8	1.9
Coumarin derivative	1	0.3	1	1.0	2	0.5
**Recreational Drugs**	**28**	**8.9**	**2**	**2.0**	**30**	**7.3**
Tetrahydrocannabinol (THC)*	18	5.8	2	2.0	20	4.8
Nicotine	9	2.9	–	–	9	2.2
Unknown drugs	1	0.3	–	–	1	0.2
**Plants and plant-based toxins**	**22**	**7.0**	**1**	**1.0**	**23**	**5.5**
Cyanogen-Glykosid (*Prunus laurocerasus*, Phaseolus spp., cherries, mirabelles*)	5	1.6	–	–	5	1.2
Grapes/raisins (Vitis ssp.)	4	1.3	–	–	4	1.0
Mushroom	3	1.0	–	–	3	0.7
Yew berries (*Taxus baccata* L.)	2	0.6	–	–	2	0.5
Lily of the valley (*Convallaria majalis*)	1	0.3	–	–	1	0.2
Beechnuts (*Fagus sylvatica*)	1	0.3	–	–	1	0.2
Unknown plant	1	0.3	–	–	1	0.2
Bark mulch	1	0.3	–	–	1	0.2
Blue green algae	1	0.3	–	–	1	0.2
Rhododendron	1	0.3	–	–	1	0.2
Ivy (*Hedera helix*)	1	0.3	–	–	1	0.2
Stagnant water	1	0.3	–	–	1	0.2
*Monstera deliciosa*	–	–	1	1.0	1	0.2
**Food leftovers/kitchen waste**	**23**	**7.3**	**–**	**–**	**23**	**5.5**
Theobromine*	11	3.5	–	–	11	2.7
Any food leftovers/kitchen waste (e.g., fish)	7	2.2.	–	–	7	1.7
Alcohol*	2	0.6	–	–	2	0.5
Caffeine*	2	0.6	–	–	2	0.5
Xylitol	1	0.3	–	–	1	0.2
**Chemicals**	**13**	**4.2**	**8**	**8.0**	**21**	**5.1**
Fertilizer	5	1.6	1	1.0	6	1.5
Didecyldimethylammonium chloride	–	–	5	5.0	5	1.2
Poison baits, incl. Parathion/sulfotep and organophosphates	3	1.0	–	–	3	0.7
Chemicals (e.g., heat pad)	2	0.6	1	1.0	3	0.7
Cleaning solution	1	0.3	1	1.0	2	0.5
Glyphosphate herbicide (round-up)	1	0.3	–	–	1	0.2
Pen/ink	1	0.3	–	–	1	0.2
**Antiparasitics**	**10**	**3.2**	**6**	**6.0**	**16**	**3.9**
Permethrin	–	–	4	4.0	4	1.0
Pyrethrum/Permethrin	3	1.0	–	–	3	0.7
Deltamethrin	2	0.6	–	–	2	0.5
Ivermectin	2	0.6	–	–	2	0.5
Permethrin + pyriproxyfen + dinotefuran	–	–	1	1.0	1	0.2
Milbemycin	–	–	1	1.0	1	0.2
Moxidectin/Imidacloprid	1	0.3	–	–	1	0.2
Fluralaner	1	0.3	–	–	1	0.2
Unknown spot-on	1	0.3	–	–	1	0.2
**Other medication**	**8**	**2.6**	**4**	**4.0**	**12**	**2.9**
**NSAIDs**	**3**	**1.0**	**2**	**2.0**	**5**	**1.2**
Ibuprofen (NSAID)	2	0.6	–	–	2	0.5
Robenacoxib (NSAID)	–	–	2	2.0	2	0.5
Diclofenac (NSAID)	1	0.3	–	–	1	0.2
**Anticonvulsants**	**1**	**0.3**	**–**	**–**	**1**	**0.3**
Phenobarbital/Potassium bromide/Imepetoin	1	0.3	–	–	1	0.3
**Other**	**4**				**12**	**1**
Metronidazole	1	0.3	–	–	1	0.2
Clozapine*	1	0.3	–	–	1	0.2
5-Hydroxytryptophan	1	0.3	–	–	1	0.2
Lisdexamfetamin	–	–	1	1.0	1	0.2
DL-mehtionine	–	–	1	1.0	1	0.2
Unknown medication	1	0.3	–	–	1	0.2
**Feces/manure**	**10**	**3.2**	**–**	**–**	**10**	**2.4**
**Nuts**	**6**	**1.9**	**–**	**–**	**6**	**1.5**
Macadamia nuts	4	1.3	–	–	4	1.0
Unknown nuts*	2	0.6	–	–	2	0.5
**Molluscicides**	**5**	**1.6**	**–**	**–**	**5**	**1.2**
Metaldehyde	5	1.6	–	–	5	1.2

*n*, number ILE-treated dogs or cats or all patients assigned to at least one of the respective toxicant groups. *Combinations of different toxicant groups or several toxicants within the same toxicant group are possible.

### 3.4. Treatment with intravenous lipid emulsion

Regardless of ILE therapy, most patients received supportive treatment, including intravenous fluids, analgesics, and antiemetics. Further decontamination and other elimination strategies (e.g., after ingestion of theobromine, walnut, coumarin derivatives, or metaldehyde) were also attempted in 244 patients prior to ILE administration (56.1%; dogs: *n* = 212, 67.7%; cats: *n* = 32, 32.0%).

Time from poisoning to first administration of ILE, reported in 243 dogs and 40 cats, was a median of 6.0 h (1.0–52.5 h) in dogs and 6.0 h (2.0–91.0 h) in cats. Time from presentation to first ILE administration was a median of 1.0 h (0.2–23.0 h) in dogs (*n* = 311) and 1.0 h (0.5–12.0 h) in cats (*n* = 97), respectively.

All dogs and cats received ILE at least once, 96 animals received a second ILE treatment (dogs: *n* = 76, 24.3%; cats: *n* = 20, 20.0%), and 26 animals had three ILE treatments (dogs: *n* = 16, 0.5%; cats: *n* = 10, 10.0%). In a few patients, ILE was administered a fourth (dogs: *n* = 6, 1.9%; cats: *n* = 3, 3.0%), fifth (dogs: *n* = 3, 1.0%), or sixth time (dogs: *n* = 1, 0.3%; Table 3).

**Table 3:** Dosages and duration of treatment with intravenous lipid emulsion (ILE) of 313 dogs and 100 cats with confirmed or suspected poisoning.

	ILE_I_		ILE_II_		ILE_III_		ILE_IV_		ILE_V_		ILE_VI_		Total		Patients	
		*n*		*n*		*n*		*n*		*n*		*n*		*n*		
DOGS	Bolus (mL/kg)	2.0 (1.0–4.0)	158	2.0 (1.0–3.1)	32	2.0 (2.0–3.0)	6	2.0	2	2.0	1	–	–	2.0 (1.0–4.0)	199	162 (51.8%)
	CRI (mL/kg/min)	0.133 (0.008–0.75)	237	0.1 (0.033–0.75)	52	0.066 (0.033–0.25)	11	0.083 (0.066–0.25)	4	0.06; 0.1	2	0.1	1	0.1 (0.008–0.75)	307	240 (76.7%)
	Duration CRI (min)	60 (15–240)	237	30 (15–240)	52	30 (20–120)	11	30 (20–60)	4	20; 30	2	20	1	45 (15–240)	307	240 (76.7%)
	ILE (mL/kg)	5.1 (1.0–66.6)	313	2.0 (1.0–19.2)	76	2.0 (2.0–19.2)	16	2.0 (2.0–15.0)	6	2.0 (2.0–2.0)	3	2.0	1	8.0 (1.5–66.6)		
CATS	Bolus (mL/kg)	2.0 (1.5–2.8)	33	1.9 (1.5–2.8)	10	1.5 (1.5–2.0)	4	1.5	2	–	–	–	–	2.0 (1.5–2.8)	49	42 (42.0%)
	CRI (mL/kg/min)	0.066 (0.03–0.26)	78	0.066 (0.05–0.25)	15	0.066 (0.066–0.25)	10	0.066 (0.066–0.1)	3	–	–	–	–	0.066 (0.03–0.26)	106	78 (78.0%)
	Duration CRI (min)	240 (15–720)	78	120 (15–240)	15	240 (15–240)	10	240 (20–240)	3	–	–	–	–	240 (15–720)	106	78 (78.0%)
	ILE (mL/kg)	14.7 (1.0–47.5)	100	2.7 (1.0–17.3)	20	15.8 (2.0–17.3)	10	17.3 (2.0–17.3)	3	–	–	–	–	15.8 (1.8–69.4)		

With up to six (dogs) or four (cats) administrations of ILE in an individual animal, a total of 199 boli of median 2.0 mL/kg (1.0–4.0 mL/kg) were given to 162 dogs (51.8%; number of boli per dog: median = 1, range: 1–4) and a total of 49 2.0 mL/kg (1.5–2.8 mL/kg) boli to 42 cats (42.0%; boli per cat: median = 1, range: 1–4). A continuous rate infusion (CRI) of ILE after the ILE bolus (*n* = 89) or without an ILE bolus (*n* = 218) was given 307 times in a total of 240 dogs (76.7%; number of CRIs administered per dog: median = 1, range: 1–6) and 106 times in 78 cats (78.0%; number of CRIs per cat: median = 1, range: 1–4). The CRI was administrated at a median rate of 0.1 mL/kg/min (0.008–0.75 mL/kg/min) over a median of 45 min (15–240 min) in dogs and at 0.066 mL/kg/min (0.03–0.26 mL/kg/min) over 240 min (15–720 min) in cats.

About three-quarters of the patients received a single ILE dose (ILE I: dogs: *n* = 237, 75.7%; cats: *n* = 80, 80.0%). In 76 dogs and 20 cats, additional ILE doses were administered. These patients received an additional two (ILE II: dogs: *n* = 60, 19.2%; cats: *n* = 10, 10.0%), three (ILE III: dogs: *n* = 10, 3.2%; cats: *n* = 7, 7.0%), four (ILE IV: dogs: *n* = 3, 1.0%; cats: *n* = 3, 3.0%), five (ILE V: dogs: *n* = 2, 0.6%), or six (ILE VI: dogs: *n* = 1, 0.3%) ILE boli or CRI. The second bolus (*n* = 32) or CRI (*n* = 52) was given at a median of 12 h (4–29 h) after the initial ILE administration, and additional ILE boli or CRIs were administered median every 12 h (4–24 h; *n* = 73) in dogs and median every 12 h (4–29 h; *n* = 20) in cats.

The total amount of ILE received was 8.0 mL/kg (1.5–66.6 mL/kg) in dogs and 15.8 mL/kg (1.8–69.4 mL/kg) in cats, which was administered median 1 dose (1–6 doses) as boli and/or CRI in dogs and median 1 dose (1–4) doses as boli and/or CRI in cats (Table 3).

### 3.5. Clinicopathologic findings

Laboratory findings (hematology and biochemistry profile) before and after ILE administration in dogs and cats are presented in Tables 4, 5. Few patients (up to 8% of dogs and 11% of cats) had follow-up analyses performed after ILE administration.

**Table 4:** Clinicopathologic findings (hematology, clinical chemistry) in 313 dogs with confirmed or suspected poisoning before (i) and after (ii) intravenous lipid emulsion (ILE) treatment.

Parameter (unit)	Reference interval	Before ILE		After ILE	
		*n*_i_	Median_i_ (range)	*n*_ii_	Median_ii_ (range)
HCT (%)	37,3–61,7	273 (87.2%)	48.2 (17.6–68.7)	17 (5.4%)	40.6 (14.5–547)
WBC (×10^9^/L)	5,05–16,76	272 (86.9%)	10.8 (2.02–30.31)	12 (3.8%)	9.3 (5.26–16.68)
Plt (×10^9^/L)	148–484	272 (86.9%)	269 (35–619)	12 (3.8%)	202 (68–403)
TP (g/L)	53.0–74.0	260 (83.7%)	64.0 (39.0–110.0)	6 (1.9%)	50.5 (40.4–68.0)
Alb (g/L)	30.9–45.0	264 (84.3%)	36.8 (21.0–80.0)	9 (2.9%)	31.5 (20.4–38.0)
Crea (μmol/L)	0–140	274 (87.5%)	84 (23–731)	22 (7.0%)	82 (42–741)
Urea (mmol/L)	2.8–8.2	265 (84.7%)	6.7 (1.8–50.0)	17 (5.4%)	4.5 (1.8–60.0)
Glu (mmol/L)	4.2–6.3	274 (87.5%)	6.1 (2.9–18.1)	21 (6.7%)	6.1 (3.6–7.8)
AST (nkat/L)	0–47	14 (4.5%)	31 (10–252)	–	–
ALT (U/L)	0–97	260 (83.1%)	45 (15–405)	6 (1.9%)	50 (41–173)
AP (U/L)	0–83	199 (63.6%)	50 (7–634)	4 (1.3%)	209 (125–589)
Bili (μmol/L)	0–3.4	130 (41.5%)	1.3 (0–33.2)	4 (1.3%)	8.6 (1.8–12.4)
Trig (mmol/L)	0.61–1.07	10 (3.2%)	0.48 (0.31–8.31)	–	–
Na (mmol/L)	143–153	257 (82.1%)	147 (122–169)	24 (7.7%)	145 (140–176)
K (mmol/L)	3.9–5.7	263 (84.0%)	4.1 (2.1–6.4)	23 (7.3%)	3.9 (2.7–6.7)
Cl (mmol/L)	99–113	257 (82.1%)	112 (90–156)	23 (7.3%)	113 (100–137)
Ca (mmol/L)	2.4–2.9	171 (54.6%)	2.8 (2.0–3.5)	6 (1.9%)	2.6 (2.3–2.7)
Ca^++^ (mmol/L)	1.12–1.4	3 (1.0%)	1.43 (1.37–1.45)	–	–

*n*, number of dogs or cats (of all 313 dogs or 100 cats in the study, respectively) in which the respective parameter was recorded. TP, total protein concentration; Alb, albumin concentration; Crea, creatinine concentration; Urea, urea concentration; Glu, glucose concentration; AST, aspartate aminotransferase activity; ALT, alanine aminotransferase activity; AP, alkaline phosphatase activity; Bili, bilirubin concentration; Trig, triglyceride; Na, sodium concentration; K, potassium concentration; Cl, chloride concentration; Ca, calcium concentration; Ca^++^, ionized calcium concentration.

**Table 5:** Clinicopathologic findings (hematology, clinical chemistry) in 100 cats with confirmed or suspected poisoning before (i) and after (ii) intravenous lipid emulsion (ILE) treatment.

Parameter (unit)	Reference interval	Before ILE		After ILE	
		*n*_i_	Median_i_ (Range)	*n*_ii_	Median_ii_ (range)
HCT (%)	30.3–52.3	85 (85.0%)	37.9 (19.7–55.1)	5 (5.0%)	40.3 (36.0–48.8)
WBC (×10^9^/L)	2.87–17.02	84 (84.0%)	8.9 (2.03–22.34)	3 (3.0%)	16.2 (14.3–23.0)
Plt (×10^9^/L)	152–600	84 (84.0%)	275 (49–632)	3 (3.0%)	185 (88–190)
TP (g/L)	57.0–90.0	81 (81.0%)	73.0 (53.0–101.0)	1 (1.0%)	67.0
Alb (g/L)	23.0–45.0	82 (82.0%)	36.0 (25.0–44.8)	2 (2.0%)	23.4; 29.0
Crea (μmol/L)	0–167	81 (81.0%)	101 (45–388)	9 (9.0%)	129 (97–406)
Urea (mmol/L)	5.2–12.5	80 (80.0%)	9.0 (4.3–57.7)	7 (7.0%)	6.8 (5.8–39.7)
Glu (mmol/L)	5.0–7.4	82 (82.0%)	6.3 (4.3–19.8)	4 (4.0%)	9.1 (5.4–17.5)
AST (U/L)	0–90	27 (27.0%)	37 (23–1,000)	2 (2.0%)	30; 31
ALT (U/L)	0–104	56 (56.0%)	54 (28–243)	–	–
AP (U/L)	0–57	22 (22.0%)	36 (18–105)	–	–
Bili (μmol/L)	0–7.0	50 (50.0%)	0.6 (0–19.0)	–	–
Trig (mmol/L)	0.19–1.17	1 (1.0%)	0.45	–	–
Na (mmol/L)	147–160	78 (78.0%)	152 (137–161)	10 (10.0%)	150 (117–154)
K (mmol/L)	3.4–5.4	78 (78.0%)	3.8 (2.4–5.5)	11 (11.0%)	4.2 (2.9–7.1)
Cl (mmol/L)	104–120	80 (80.0%)	117 (78–127)	11 (11.0%)	116 (90–127)
Ca (mmol/L)	2.2–2.98	64 (64.0%)	2.7 (2.2–3.2)	–	–
Ca^++^ (mmol/L)	1.12–1.4	2 (2.0%)	1.33; 1.54	–	–

*n*, number of dogs or cats (of all 313 dogs or 100 cats in the study, respectively) in which the respective parameter was recorded. TP, total protein concentration; Alb, albumin concentration; Crea, creatinine concentration; Urea, urea concentration; Glu, glucose concentration; AST, aspartate aminotransferase activity; ALT, alanine aminotransferase activity; AP, alkaline phosphatase activity; Bili, bilirubin concentration; Trig, triglyceride; Na, sodium concentration; K, potassium concentration; Cl, chloride concentration; Ca, calcium concentration; Ca^++^, ionized calcium concentration.

### 3.6. Effect of ILE

The effect of ILE was defined as the resolution or improvement of the intoxication-associated clinical signs after ILE administration. The clinical condition either resolved or improved (dogs: *n* = 234, 74.8%; cats: *n* = 54, 54.0%), was unchanged (dogs: *n* = 66, 21.1%, cats: *n* = 42; 42.0%), or deteriorated (dogs: *n* = 13, 34.2%; cats: *n* = 4, 4.0%) after ILE I. After ILE II, an improvement was observed in 64/76 dogs (84.2%) and 13/20 cats (65.0%), whereas 11 dogs (14.5%) and 7 cats (35.0%) were unchanged and 1 dog (1.3%) deteriorated. Overall, the administration of ILE was followed by a clinical improvement in 73.6% (*n* = 304) of the patients and a deterioration in 4.4% (*n* = 18). In 22% (*n* = 91) of the animals, ILE appeared to not have any substantial effect on the clinical signs (Table 6).

**Table 6:** Effect of treatment with intravenous lipid emulsion (ILE) in 313 dogs and 100 cats with confirmed or suspected poisoning.

	Effect after ILE	Total		Improved clinical condition		Unchanged clinical signs		Worsened clinical condition	
		*n*	%	*n*	%	*n*	%	*n*	%
Dogs	ILE I	313	100.0	234	74.8	66	21.1	13	4.2
	ILE II	76	24.3	64	84.2	11	14.5	1	1.3
Cats	ILE I	100	100.0	54	54.0	42	42.0	4	4.0
	ILE II	20	20.0	13	65.0	7	35.0	–	–

ILE I, first time of ILE administration; ILE II, second time of ILE administration.

### 3.7. Adverse effects of ILE

Adverse effects presumed to be associated with the administration of ILE occurred in 20/313 dogs (6.4%) and 4/100 cats (4.0%) in 24/413 patients (5.8%) after ILE administration (Table 7). Such effects were presumed to be adverse effects if occurring mostly within 0.5–4.0 h or if the clinical signs (regardless of being consistent with those expected with the respective poisoning) were seen within 24 h but not prior to ILE use. These included the temporary loss of consciousness (comatose state, recumbency; dogs: *n* = 6, cats: *n* = 1; 7/413, 1.7%) or reduced level of consciousness (somnolence; dogs: *n* = 2; cats: *n* = 1; 3/413, 0.7%). Bradycardia (dogs: *n* = 2, cats: *n* = 1), hyperthermia (dogs: *n* = 3), vomiting (dogs: *n* = 3), diarrhea (dogs: *n* = 3), respiratory distress (apnea, dyspnea, and tachypnea; dogs: *n* = 2; cats: *n* = 1), or deterioration of the general behavior (dogs: *n* = 3) were also recorded (each in 3/413 animals, 0.7%). Facial swelling as an allergic reaction (dogs: *n* = 1), ataxia (dogs: *n* = 1), and thrombophlebitis (cats: *n* = 1) were also observed (each in 1/413 animals, 0.2%).

**Table 7:** Adverse effects of intravenous lipid emulsion (ILE) in 20/313 dogs (6.4%) and 4/100 cats (4.0%) to treat confirmed and suspected poisoning.

	Adverse effect	*n*	Adverse effect ILE associated	Amount of ILE (mL/kg)	Outcome
Dogs	Loss of consciousness	6	Very probable (6)^a^^,^^b^^,^^c^^,^^d^	15.0 (2.0–66.6)	Survived
	Hyperthermia	3	Possible (3)^e^^,^^f^^,^^g^	5.9 (8.0–30.0)	Survived
	Vomiting	3	Very probable (3)^a^^,^^c^^,^^h^	30.3 (2.2–66.6)	Survived
	Diarrhea	3	Possible (2)^a^^,^^i^ Very probable (1)^j^	30.3 (11.9–31.5)	Survived
	Reduced level of consciousness	2	Possible (2)^j^^,^^k^	9.0; 15.0	Survived
	Bradycardia	2	Very probable (1)^l^ Possible (1)^m^	9.4; 11.5	Survived
	Apnea/Dyspnea	2	Possible (2)^a^	5.1; 15.0	Euthanized (1) survived (1)
	Reduced/worsening of general behavior	3	Very probable (2)^a^^,^^n^ Possible (1)^a^	16.2 (11.5–30.3)	Survived
	Facial swelling, suspicion of an allergic reaction	1	Very probable^a^	2.0	Survived
	Ataxia	1	Very probable^a^	30.3	Survived
Cats	Tachypnea	1	Very probable^a^	47.5	Survived
	Thrombophlebitis	1	Very probable^o^	15.8	Survived
	Semicomatose state	1	Very probable^p^	2.0	Survived
	Somnolence	1	Very probable^a^	11.9	Survived
	Bradycardia	1	Very probable^a^	11.9	Survived
Total		24		11.88 (2.0–66.6)	Survived (23) euthanized (1)

a Unknown toxicant (*n* = 11).

b Chemical (pen, ink; *n* = 1).

c Plant and plant-based toxin (grapes/Vitis sp.; *n* = 2).

d food leftovers/kitchen waste (meat and onions; *n* = 1).

e Potentially tremorgenic mycotoxins (garbage; *n* = 1).

f Recreational Drug (nicotine; *n* = 1).

g Food leftovers/kitchen waste (caffeine; *n* = 1).

h Food leftovers/kitchen waste (meat and onions; *n* = 1).

i Other medication (diclofenac; *n* = 1).

j Recreational Drug (THC = tetrahydrocannabinol; *n* = 1).

k Nuts (macadamia nuts; *n* = 1).

l Food leftovers/kitchen waste (sausage and cherries; *n* = 1).

m Antiparasitic (permethrin; *n* = 1).

n Rodenticides (coumarine derivative; *n* = 1).

o Rodenticides (α-chloralose; *n* = 1).

p Rodenticides (unknown rodenticide; *n* = 1).

*n*, numbers of animals with the respective adverse effect.

Adverse effects were observed after administration of a median of 11.9 mL ILE/kg (2.0–66.6 mL/kg), including ILE boli at a median of 1.5 mL/kg (2.1–2.0 mL/kg) in 12 dogs and a CRI at a median of 0.25 mL/kg/min (0.008–0.37 mL/kg/min) over median 60 min (20–720 min) in 17 dogs and 4 cats. A higher total amount of ILE was given to dogs that experienced adverse effects (median: 11.7 mL/kg; range 2.0–66.7 mL/kg) than to dogs without adverse effects of ILE treatment (median: 7.5 mL/kg; range: 1.5–61.5 mL/kg; *p* = 0.042). In cats, there was no significant difference between these two groups (total ILE volume with adverse effect: median: 13.9 mL/kg; range: 2.0–47.5 mL/kg vs. without adverse effects: median 15.9 mL/kg; range: 1.9–69.4 mL/kg; *p* = 0.695). One dog with suspected poisoning with an unknown toxicant experienced respiratory arrest and was euthanized. In all other patients, adverse effects of ILE therapy were transient, and these patients survived (23/24, 95.8%) without severe long-term consequences. Some patients were discharged despite still having mild signs of questionable adverse effects such as diarrhea (*n* = 2) after ingestion of diclofenac and THC or hyperthermia (*n* = 2) after ingestion of caffeine or nicotine.

Few patients that experienced adverse events underwent decontamination efforts (5/24, 20.8%; all dogs) before ILE administration. This included induced emesis (3/5), gastric lavage (1/5), and gastrointestinal lavage (1/5). Among those patients where decontamination was not previously attempted, some dogs had already vomited (4/19), and others received activated charcoal (7/19) or intestinal lavage (1/19). Omitting decontamination had a higher odds ratio (OR) for the development of adverse effects after ILE (*p* < 0.001; OR 6.06; 95% confidence interval: 2.2–16.6).

Adverse effects occurred during or after ILE administration for the treatment of poisoning with an unidentified toxicant (*n* = 9), rodenticides, food leftovers/kitchen waste, or recreational drugs (*n* = 3 each), chemicals, toxic plants, medication, nuts, or antiparasitics (*n* = 1 each; Table 7).

### 3.8. Outcome

Most patients (367/413, 88.9%; dogs: *n* = 273, 87.2%; cats: *n* = 94, 94.0%) were hospitalized for a median of 1 day (dogs: median 0.9 days, range 0.1–6.0 days; cats: median 2.0 days, range 0.1–5.0 days). Most (395/413, 95.6%) of those ILE-treated patients survived to discharge from the hospital (dogs: *n* = 304, 97.1%; cats: *n* = 91, 91.0%). Thirteen patients (3.1%) were euthanized (dogs: *n* = 5, 1.6%; cats: *n* = 7, 7.0%) and six patients (1.5%) died (dogs: *n* = 4, 1.3%; cats: *n* = 2, 2.0%).

Overall time to clinical improvement after presentation was a median 7.0 h (1.0–230.0 h; *n* = 388). Dogs recovered faster (median: 7.0 h, range: 1.0–230.0 h; *n* = 298) than cats (median: 11.0 h, range: 2.0–65.0 h; *n* = 90; *p* < 0.001). Time to improvement correlated mildly with ILE total dose in dogs (*r* = 0.207; *p* < 0.001), but not in cats (*p* = 0.925). Longer recovery times of 4–6 days occurred in dogs with walnut (*n* = 3), oleander, tetrahydrocannabinol (THC), permethrin, alpha-chloralose, or metronidazole poisoning, or exposure to an unidentified substance (each *n* = 1). During hospitalization, no clinical improvement could be documented in 18/413 patients (4.4%; dogs: *n* = 10, 3.2%; cats: *n* = 8, 8.0%), or a statement regarding the clinical improvement was not possible based on the patient medical records (7/413, 1.7%; dogs: *n* = 5, 1.6%; cats: *n* = 2, 2.0%).

## 4. Discussion

Over the 5 years period of this retrospective investigation, ILE was administered in 313 dogs and 100 cats. This represents about half (52%) of the animals presented with poisonings during that time period (413/800), which is the largest series of dogs and cats with ILE treatment for poisonings compared to previous reports including less than 20 cats and 9 dogs, respectively (1, 9, 27).

Seizures were a frequent reason for presentation to the emergency service (dogs: *n* = 24; cats: *n* = 30). Some of the affected animals presented in status epilepticus (dogs: *n* = 13; cats: *n* = 11) or postictal state (dogs: *n* = 1; cats: *n* = 1), while others were already sedated (dogs: *n* = 8; cats: *n* = 4). These difficulties in controlling seizures, similar to paralysis and coma, can result in complications, such as aspiration pneumonia or renal injury carrying a high mortality rate. These cases are indications for the use of ILE if lipophilic toxicants are presumed or confirmed (1, 28, 35). Still, administration of a 20% lipid solution to treat lipophilic drug poisoning is an off-label use of ILE and must be decided on an individual basis weighing the expected benefits against potential (neurological) side effects (12, 32). The key parameter to determine the indication for ILE application is log P, which is the logarithm of the partition coefficient calculated for the respective substance from a two-phase system (octanol and water) and reflects its lipophilicity. Substances with a log *p* > 1.0 accumulate in lipophilic solvents as a result of the concentration gradient that is created between the lipophilic and hydrophilic phases (“lipid sink”), and can thus be removed from the tissue (particularly heart, brain, and musculature) by means of ILE therapy (6, 12). The current “lipid shuttle” theory includes the transport to the liver and the degradation and hepatic and/or renal excretion of the lipophilic toxicants after their removal from the plasma pool (28).

Permethrin (log *p* = 6.24), THC (log *p* = 6.1), moxidectin (log *p* = 5.3), ivermectin (log *p* = 4.37), carprofen (log *p* = 4.09), ibuprofen (log *p* = 3.5), and lidocaine (log *p* = 1.81) are examples for substances that are suitable for removal via ILE therapy^1^ (8, 17, 29, 40–43). In contrast, the use of ILE is not useful with metaldehyde (log *p* = 0.12), ethylene glycol (log *p* = −1.36), or xylitol (log *p* = −2.56) poisoning^2^,^3^,^4^. However, unsuccessful application of ILE has also been described for poisonings with substances with high log *p* values such as ivermectin (11) or bromethalin and organophosphates (27). Given the variation in the log p value reported for metaldehyde in the literature,(see footnote 2) the assumed lipophilicity (with log *p* = 1.1) justifies the use of ILE for the treatment of metaldehyde poisoning. Assuming that metaldehyde is of rather low lipophilicity (with log *p* = 0.12), administration of ILE would presumably be ineffective with metaldehyde poisoning. Nevertheless, a beneficial effect has been reported in canine and feline metaldehyde poisoning for ILE combined with other supportive measures (44, 45). In this study, central nervous system signs were not improved after administration of several anticonvulsants but treatment including ILE led to a remarkable improvement of the tonic–clonic activity and complete resolution of the clinical sign (44). Thus, the effectiveness of ILE in metaldehyde toxicosis remains unclear, and hemodialysis would be the preferred approach to elimination in these cases (46). In the present study, the beneficial effect of ILE in cases of metaldehyde poisoning could not be reliably attributed to ILE administration alone, although 4/5 dogs (80%) were clinically unremarkable at discharge from the hospital. However, one of those dogs was euthanized due to fulminant aspiration pneumonia after ingestion of metaldehyde.

There were only few individual cases (<8%) in which post-treatment biochemical parameters were recorded allowing for a comparison of the values before and after treatment to evaluate the effect of ILE. Laboratory values important after ILE application, such as triglycerides or blood gas analysis, were not recorded. Thus, given the small number of patients, conclusions about an effect of ILE based on laboratory parameters is not possible.

Early administration of ILE after toxicant ingestion is key for therapeutic success. In the present study, ILE treatment was initiated after a median of 6.0 h after ingestion of or contact with the toxicant, whereas the first ILE bolus or CRI was given at a median of 1.0 h after the presentation, showing that the delay was mostly due to the time from poisoning to presentation. Still, this approach allowed a fast recovery (within a few hours) in most cases, whereas longer recovery periods in dogs (up to 6 days) can be explained by neurotoxic effects and a postictal state (e.g., with walnuts, THC, metronidazole, permethrin, or alpha-chloralose).

Generally, several different doses, dosing intervals, and durations between applications were used. ILE boli were mostly administered at 2.0 mL/kg in both dogs (109/199, 54.8%) and cats (20/49, 40.8%), whereas a dose of 1.5 mL/kg was administered in some dogs (74/199, 37.2%) as used or recommended by others (13, 27, 35). While CRI dosing of 0.066 mL/kg/min (81/106, 76.4%) over 240 min (65/106, 61.3%) seemed to be preferred in cats in the clinic, consistent doses of ILE were administered in only about one-third of the dogs. Rates of 0.25 mL/kg/min (118/307, 38.4%) over 60 min (99/307, 32.2%) were mostly used in large dogs and 0.066 mL/kg/min (120/307, 39.1%) over 240 min (42/307, 13.7%) was used in smaller dogs and those with a cardiac condition or unstable cardiovascular status (15). In cats, most authors follow the typical dose in dogs of 1.5 mL/kg (bolus) with a CRI of 0.25 mL/kg/min over 30–60 min (1, 9, 43, 47, 48). However, low-dose ILE treatment strategies (1.5 mL/kg bolus followed by 0.25 mL/kg/min over 3 min, 0.025 mL/kg/min over 360 min, or 1.5 mL/kg followed by 0.025 mL/kg over 3–9 h) are also reported to be ineffective or potentially effective (45, 49), but were not effective within 2–12 h in our study. Two to six different ILE administrations were mostly given 12 h apart (52/95, 54.7%).

Most patients experienced treatment success as assessed based on the clinical status, after a single dose of ILE (76.8%), whereas clinical signs continued to improve after a second (17%), third (4.1%), or fourth to sixth ILE dose (2.2%) in some patients. Repeat dosing of ILE is not routinely performed, but appears safe (with ≤2 mL/kg per dose) and might offer additional benefit. However, our results support the recommendation to discontinue ILE treatment if an effect is not seen after 2–3 doses (35).

Total ILE doses of 6.1–33.0 mL/kg (16.5 mL/kg in most case series) are reported in dogs and 15–18 mL/kg (up to 31.5 mL/kg) in cats (1, 8, 15–17, 27, 34, 43, 50, 51), which is largely consistent with the ILE doses in our study (medians: 8.0 mL/kg/dog and 15.8 mL/kg/cat). ILE treatment has a high margin of safety within recommended and commonly used doses, with an estimated IV LD_50_ in rats of 67 mL/kg (31), which justifies the use of larger maximum doses than reported. Still, application of up to twice the dose recommended by others should be reserved for individual severe cases. Following the recommendations of Robben and Dijkmann, the total maximum dose should be 10–12 mL/kg over a period of 30 min (35). However, whether the clinical improvement of a patient can be attributed to a positive effect of ILE treatment cannot be verified, particularly with repeated dosing, because hepatic metabolization and subsequent elimination of the toxicant contribute to its removal from the body similar to other supportive measures irrespective of ILE therapy.

In this retrospective evaluation, ILE was given pre-emptively in some patients (e.g., after NSAID overdose absent any clinical signs or clinicopathologic abnormalities) which count towards the population of animals with an unchanged condition (ILE I: 26%; ILE II: 19%) after ILE administration. On the other hand, failure of ILE to have positive effects could also be explained by the lack of decontamination, representing another limitation of this retrospective evaluation. Additionally, in the absence of lipophilicity, toxicant elimination by ILE administration is not expected for toxicants such as α-chloralose, coumarin derivatives, metronidazole, and theobromine, and is questionable for macadamia nuts, walnuts, and metaldehyde. However, the decision to use ILE was at the discretion of the clinician and was often made based on the presence of severe neurological sings. Also, the contribution of simply time and physiological metabolism/excretion, unknown substances and doses exposed to in some cases, further supportive care, ILE administration, and/or in some cases a potential “lipid shuttle” created by the lipophilic drug propofol used for general anesthesia (log *p* = 3.79^5^) on the clinical improvement of individual animals cannot be distinguished. This challenge is further supported by a more rapid improvement observed in cats with permethrin intoxication receiving ILE therapy in a prospective randomized study (1), but the lack of a beneficial effect of ILE in rabbits with ivermectin poisoning in another study raised concerns over the use of ILE as a general antidote or first-line treatment and recommended ILE only in life-threatening situations or with financial constraints (2).

Adverse effects of ILE were classified as “very probable” if occurring immediately after ILE administration (*n* = 20), or as “possible” for any effect that could have also been caused by the toxicant, but the patient did not show this clinical sign to the same extent prior to ILE therapy (*n* = 11). However, progressive clinical signs caused by the toxicant (e.g., progressive bradycardia or somnolence in poisoning with THC, hyperthermia due to metaldehyde poisoning) cannot be reliably distinguished from the variance of clinical signs or possible sequelae (e.g., postictal) in all cases. Adverse effects of ILE considered as very likely occurred in a few cases (*n* = 12) of poisoning with an unidentified toxicant, rodenticides, food waste, chemicals, or plants. Progression of clinical signs of poisoning such as recumbency, comatose state, vomiting, dyspnea or apnea, somnolence, and bradycardia are also challenging to distinguish from signs of nonspecific worsening after ingestion of an unknown toxicant or without decontamination measures. For example, somnolence could have also been induced by recreational drugs (THC, *n* = 1) or hyperthermia by tremorgenic mycotoxins (contained in the garbage, *n* = 1), recreational drug ingestion (nicotine, *n* = 1), or ingestion of food leftovers/kitchen waste containing caffeine (*n* = 1). Similarly, diarrhea could have been caused by ingestion of an NSAID (diclofenac, *n* = 1) or recreational drug (THC, *n* = 1), sudden reduced consciousness and cardiovascular status may have been due to macadamia ingestion (*n* = 1), or progressive bradycardia resulting from permethrin ingestion (*n* = 1) rather than presenting adverse effects of ILE. Erring on the side of caution and minimizing the risk of side effect neglect, all clinical deteriorations that could have possibly been linked to ILE administration were assumed and included as such. Thus, while the study suggests ILE therapy to be successful in some cases, another important conclusion – given the presumably overestimated temporary side effects in about 6% of the patients – is that ILE appears safe in dogs and cats as adverse effects were not observed in >94% of ILE-treated dogs and cats. In line with this, standardized documentation of the clinical course is lacking due to the retrospective nature of this study, and medical record entries are more likely for adverse effects that occurred than steps of clinical improvement.

In veterinary medicine (VM), similar complications to human medicine (HM) are described for ILE treatment of poisoning and total parenteral nutrition (TPN). These include cardiovascular complications including asystole, cardiac arrest, pulmonary microembolism, and lower rates of returning spontaneous circulation after resuscitation [HM: (18, 52, 53); VM: (19)] and pulmonary adverse effects such as acute respiratory distress syndrome (ARDS) [HM: (20, 54); VM: (55, 56)] and hypoxia [VM: (57)], pulmonary hypertension [VM: (58, 59)], or a ventilation/perfusion mismatch [HM: (22); VM: (23, 60)] in HM and experimental animal models. Dyspnea could be caused by a reduced surfactant function with ARDS [VM: (61)] and hematologic abnormalities resulting from ILE administration include intravascular hemolysis [HM: (62)], anemia [VM: (63)], thrombocytopenia [HM: (24); VM: (25)], and disseminated intravascular coagulation [HM: (38)]. Whether the risk of lipid emboli remains the same or increases with each ILE administration warrants further study [HM: (32, 64)]. Neurologic signs (e.g., weakness, seizures) can result particularly with large-droplet emulsions, requiring the use of an in-line filter [VM: (26–28), HM: (65)]. Still, even parenteral nutrition using lipid solutions can lead to focal or generalized seizures via cerebral endothelial and intravascular lipid deposition, and this mechanism could contribute to reduced consciousness or semi-comatose states also with ILE therapy (VM: 29). Hypertriglyceridemia is reported both in HM (20, 30, 38) and VM (31) and can cause intravascular lipid deposition in the liver, kidneys, brain, and other organs. Lipid overload syndrome can explain fever, hepatomegaly, splenomegaly (or rarely pancreatitis), anemia, leukopenia, thrombocytopenia, coagulopathy, hemolysis, and hepatic dysfunction [HM: (32, 52, 66); VM: (33)]. Rarely, corneal lipidosis has been described in cats [VM: (34, 50)]. Acute anaphylactoid reactions resulting in pruritus, dermal blisters, urticaria, diffuse erythema, tachypnea, pyrexia, nausea, vomiting, diarrhea, systemic hypotension, cyanosis, and severely reduced general condition can occur after TPN or ILE as treatment of poisoning [VM: (1, 35), HM: (36, 67–69)]. An increased risk of infection caused by neutropenia and lymphopenia [VM: (70)] and the development of systemic inflammatory response syndrome (SIRS) or sepsis (after application of contaminated emulsions) are also reported with TPN in humans and with ILE use in laboratory animal studies including rats and rabbits [VM: (71, 72); HM: (73)], and toxicants could also be increasingly absorbed from the gastrointestinal tract with ILE therapy [VM: (74, 75)]. Possible complications of ILE administration that have only been reported in HM to date include acute kidney injury [HM: (37, 76)], metabolic acidosis [HM: (38)], and vascular occlusive conditions (e.g., phlebitis, thrombosis, and priapism) [HM: (39, 77, 78)]. Some of these adverse effects were documented in this retrospective evaluation: a temporary loss of consciousness (comatose state, recumbency) or reduced level of consciousness (somnolence), bradycardia, hyperthermia, vomiting, diarrhea, respiratory distress, reduced general behavior, facial swelling (allergic reaction), ataxia, and thrombophlebitis.

Hypervolemia is a possible concern with a rate of 15 mL/kg/h in cats, particularly with (occult) cardiac disease. Thus, the desired amount of ILE is preferentially administered over a longer period [e.g., 4 h in this study; (50)]. Maximum doses for ILE in human parenteral nutrition are set at 12.5 mL/kg/d by the US Food and Drug Administration (FDA) (79) – 5–10 mL/kg/d is listed on the package insert for Lipofundin 20% N^6^ – and a maximum of 10 mL/kg/d has been recommended in VM (12) despite tolerance for much higher dosages for acute treatment of poisoning (30). However, adverse effects also occurred after a median ILE of 11.9 mL/kg (bolus of a median of 1.5 mL/kg followed by a CRI of 0.25 mL/kg/min over 60 min), showing that adverse effects can also be seen at lower doses of ILE.

The occurrence of lipid overload syndrome, however, not only depends on the infusion rate and total dose but is also affected by the composition of the lipid emulsion (80). Fat overload syndrome or microemboli are described almost exclusively with the use of soybean oil products (18, 33). It is possible that the increased levels of proinflammatory omega(ω)-6 fatty acids contained therein lead to impaired immune function. Intralipid, which is commonly used for TPN, is 100% soybean oil and contains high levels of the long-chain linoleic acid (C18:2) (66), which can result in decreased monocyte function and bactericidal effects on neutrophilic granulocytes, potentially resulting in a higher rate of the complications detailed above (81, 82). In contrast, Lipofundin – as used in the case series – is half coconut oil and half soybean oil. The larger proportion of medium-chain triglycerides (MCT) is excreted more rapidly and tends to cause less hyperlipidemia when infused slowly (83). With fish oils (ω-3 fatty acids), it was traditionally assumed that the complications seen with soybean oil emulsions do not occur as the residence time in the systemic circulation is too short given the increased lipolysis and accelerated triglyceride clearance. Fat overload syndrome was first described in 2013 in a 2 years-old girl (33) after rapid application of SMOFlipid (20% soy oil, MCT, olive and fish oil-based lipid emulsion) and – 2 years later – was followed by a case report of an adult 72 years-old man (84).

Finding that adverse effects of ILE occur predominantly (up to 79%) with the omission of prior decontamination (*n* = 169) underscores the importance of these measures before initiation of ILE therapy (35) as toxicants still present in the gastrointestinal tract can be increasingly absorbed due to the increased plasma lipophilicity (74, 75). Complete decontamination was performed in only 59% of the patients, although it is recommended in addition to patient stabilization and should be prioritized in patients with poisoning (85–88). Thus, the lack of decontamination resulting in increased absorption of the toxicant could have contributed to the clinical deterioration in some cases (89, 90) given that dogs and cats were more likely to experience adverse effects without prior decontamination. However, the potential benefits must be carefully weighed against possible risks and contraindications, such as too long of a delay between toxicant exposure and hospital admission in individual patients, presenting with impaired consciousness, seizures, impaired swallowing, or other risk factors for aspiration pneumonia. If toxic effects are not expected (e.g., after ingestion of small quantities of theobromine), decontamination and elimination measures may not be required. The high survival rate of 96% in this study likely also reflects success after the earliest possible intervention in poisoning cases as supported by similar survival rates in other case series of poisonings in cats [83–86% (91–93)] and dogs [83–89% (94–97)]. Patients with poisonings that were presented to our clinic during the same time period, but were not treated with ILE, had a similar survival rate of 95% (311/321 = 96.9% in dogs and 56/66 = 84.8% in cats). Because most poisoned animals survive with intensive and immediate veterinary care, clinical improvement within a median of 7 h may not necessarily be linked to ILE therapy.

The retrospective design of our study carries some risk of bias or lack of patient information. Also, conclusions as to the time interval at which clinical improvement can be expected or ILE administration should be recommended or even repeated are limited. Further prospective investigations, including clinical controls, standardized treatment protocols, objective evaluation criteria (e.g., Glasgow coma scale) and clinical (electrocardiography (ECG) and blood pressure monitoring) as well as clinicopathologic variables (blood gas analysis, blood biochemistry, including triglycerides and lactate, coagulation testing, and urinalysis) are now needed to establish evidence-based recommendations for ILE treatment and monitoring in dogs and cats. Some of these parameters were evaluated in this study, but conclusions are limited without controls. Useful information was provided for possible adverse effects of ILE. Given that many dogs and cats received ILE preemptively and standardized protocols for the treatment and monitoring of patients with presumed or confirmed exposure to hydrophobic poisoning agents were lacking, the treatment efficacy of ILE has to be carefully interpreted. Although the study suggests that ILE is safe to use in small animal patients, the possibility of adverse effects and limited evidence of clear benefits preclude the recommendation to use ILE routinely in the treatment of poisoning cases. Rather, its indication should be carefully considered on an individual basis.

## 5. Conclusion

Reviewing 313 canine and 100 feline poisoning cases treated with ILE beneficial effects ascribed to the administration of ILE are presumed based on the clinical impression, but their quantification remains challenging. The clinical deterioration due to ILE adverse effects cannot be reliably differentiated from toxicant-associated complications, but the rate of adverse effects is suggested to be generally low (<6%). Lack of adverse effects in >94% of ILE-treated patients confirms a wide window of safety at the recommended doses (1.5 mL/kg bolus followed by 0.25 mL/kg/min over 30–60 min in dogs and cats or a CRI of 0.066 mL/kg/min over 240 min in cats). ILE-treated cases also had a high survival rate, but apnea requiring euthanasia in one case cannot be excluded as an adverse effect of ILE administration.

## Data availability statement

The raw data supporting the conclusions of this article will be made available by the authors, without undue reservation.

## Ethics statement

Ethical approval was not required for the study involving animals in accordance with the local legislation and institutional requirements because Ethical review and approval was not required for the animal study because data were only retrospectively analyzed.

## Author contributions

CM: Conceptualization, Formal analysis, Data curation, Writing – original draft. RH: Formal analysis, Methodology, Validation, Writing – review & editing. DK: Data curation, formal analysis, review. RD: Formal analysis, Methodology, Validation, Writing – review & editing, Conceptualization, Funding acquisition, Project administration, Resources, Supervision.

## Footnotes

^1^Drugbank (2023). https://go. drugbank.com/drugs [accessed: May 14, 2023].

^2^ National Center for Biotechnology Information (NCBI). PubChem Compound Summary for CID 61021, Metaldehyde (2023). https://pubchem.ncbi.nlm.nih.gov/compound/Metaldehyde [accessed: May 14, 2023].

^3^ NCBI. PubChem Compound Summary for CID 174, Ethylene Glycol (2023). https://pubchem.ncbi.nlm.nih.gov/compound/Ethylene-Glycol [accessed: May 14, 2023].

^4^NCBI. PubChem Compound Summary for CID 6912, Xylitol (2023). https://pubchem.ncbi.nlm.nih.gov/compound/Xylitol [accessed: May 14, 2023].

^5^NCBI. PubChem Compound Summary for CID 4943, Propofol (2023). https://pubchem.ncbi.nih.gov/compound/Propofol. [accessed: May 14, 2023].

^6^Medikamio. Lipofundin 20% N B. Braun (2023). https://www.medikamio.com/de-de/medikamente/lipofundin-20-n/pil [accessed: 22.05.2023].
